# Comparative Histopathological Evaluation of Gingival Tissue Reactions to Chlorhexidine-Coated and Uncoated Silk Sutures in Male Rats: Comparative Tissue Reactions to Chlorhexidine-Coated and Uncoated Silk Sutures

**DOI:** 10.31661/gmj.v13iSP1.3577

**Published:** 2024-12-31

**Authors:** Mohammad Reza Keshavarz, Reyhaneh Ebrahimi, Samira Sadat Abolmaali, Shima Torabi Ardekani, Omid Koohi-Hosseinabadi, Hamid Reza Arabion, Nader Tanideh, Fariborz Nowzari

**Affiliations:** ^1^ Department of Oral and Maxillofacial Surgery, School of Dentistry, Shiraz University of Medical Science, Shiraz, Iran; ^2^ Department of Periodontology, School of Dentistry, Shiraz University of Medical Sciences, Shiraz, Iran; ^3^ Pharmaceutical Nanotechnology Department and Center for Nanotechnology in Drug Delivery, Shiraz University of Medical Sciences, Shiraz, Iran; ^4^ Department of Oral and Maxillofacial Pathology, School of Dentistry, Shiraz University of Medical Sciences, Shiraz, Iran; ^5^ Center of Comparative and Experimental Medicine, School of Medicine, Shiraz University of Medical Sciences, Shiraz, Iran; ^6^ Stem Cell Technology Research Center, Shiraz University of Medical Sciences, Shiraz, Iran

**Keywords:** Silk Sutures, Chlorhexidine, Tissue Reaction, Histopathology, Rat Model

## Abstract

**Background:**

Surgical sutures play a crucial role in wound healing and inflammation management. Sutures coated with chlorhexidine are designed to provide secondary antimicrobial protection. However, the impact of these chlorhexidine-coated silk sutures on immediate tissue reactions, compared to ostensibly inert suture materials, has not been extensively investigated. This study aims to compare tissue responses caused by the chlorhexidine coated silk sutures or uncoated silk suture in rats, as a guide to potential benefits clinically.

**Materials and Methods:**

In this study, 4-0 silk sutures were coated with 3% chlorhexidine using Eudragit RL polymer. A total of eighteen male Sprague-Dawley rats (10-wk-old, 200±20 gr) were randomly divided into three groups, with six in each group. Animals were anesthetized using ketamine hydrochloride and xylazine. A 5-mm incision was made on the keratinized gingiva between their right and left upper second premolars at both sides using a scalpel blade. The left flap was closed using chlorhexidine-coated sutures, while the right one was sutured with standard ones. On the 3rd, 5th, and 7th postoperative days biopsies from the suture sites were obtained for pathological examination after euthanasia. After determining normality and homogeneity of variance, inflammation was analyzed using the Kruskal-Wallis test for nonparametric data; formation of fibrous and granulation tissue was assessed with a chi-square test. A P-value0.05 was considered significant as recommended.

**Results:**

Histopathological evaluation of tissue extracted on the 3rd, 5th, and 7th days showed no statistically significant difference in tissue inflammation, granulation, or fibrous connective tissue accumulation between chlorhexidine-coated silk sutures and uncoated silk sutures.

**Conclusion:**

The study results indicated that chlorhexidine-coated silk sutures induced tissue responses comparable to those of uncoated silk control sutures. These data suggest that, although the release of chlorhexidine in oral solutions may be achieved with these sutures, potentially aiding in the effective inhibition of bacterial growth during wound healing, they do not demonstrate anti-inflammatory effects or cause any adverse tissue responses.

## Introduction

Sutures are among the most basic instruments in surgical practice, used to close wounds and aid the wound healing process. The choice of suture material can significantly influence the course of healing, affecting tissue reactions and infection rates, and ultimately impacting surgical success [1]. The interaction of sutures with tissue is a complex process that depends on the suture material itself, any coating applied to it, and the biological environment in which it resides [2]. Sutures that are biocompatible and able to withstand the oral bacterial load have been used for suturing tissues located in the mouth, minimizing potential complications [3]. Silk sutures have consistently been favored for their versatility and established reliability, but they do have some drawbacks. Silk sutures are regarded as filaments that can be readily colonized by bacteria and, in some cases, may cause inflammatory reactions [4]. This is especially important in the oral cavity, which has a higher microbial flora density than typical skin incisions [4]. Medical silk is also recognized for triggering an intensified inflammatory response to pathogens, attributed to its molecular properties [5]. These problems have prompted research into improved suture materials which are intended to address most of these concerns.

In this context, one of the most promising approaches is the use of suture coatings with chlorhexidine, an effective antimicrobial and anti-inflammatory agent [6][7]. Chlorhexidine is well-known in the medical field for its effectiveness in reducing bacterial presence and preventing infections [8]. Since chlorhexidine can be used for suture coating, it offers a unique method to achieve a localized antimicrobial effect that may reduce the incidence of surgical site infections [9]. Although, these coated sutures are currently under research for their effect on tissue response.

The body’s response to sutures involves a series of inflammatory reactions, tissue remodeling, and, ultimately, wound healing. Essentially, the most critical of these responses are inflammatory reactions. Aberrant inflammation can result in delayed healing, fibrosis, and granulation tissue formation [2].

Several reports suggest that these sutures may reduce bacterial colonization and infection, which is particularly useful when applied to contaminated surgical fields where infections are highly likely [10][11]. However, reports on the wound-healing effects of chlorhexidine are also highly controversial, with many studies suggesting that even low concentrations of chlorhexidine can exhibit cytotoxic effects, potentially leading to delayed wound healing [12][13]. The degree of inflammation and the presence of granulation tissue are key indicators of how well the body tolerates a particular suture material [14]. Therefore, it is essential to evaluate these factors to understand the biocompatibility and clinical efficacy of these sutures for use in surgical procedures.

Many studies focus on the antibacterial effects of chlorhexidine-coated sutures without considering tissue reactions. In this regard, this study investigated the influence of chlorhexidine-coated sutures on tissue responses, including inflammation and fibrous tissue formation.

Understanding these effects is essential for assessing the overall safety and usefulness of chlorhexidine-coated sutures in medical practice. This study aimed to address this lack of information by comparing the tissue reactions following suture insertion between chlorhexidine-coated silk sutures and standard uncoated silk ones, in a rat model. Our research specifically examines inflammatory responses, the presence of granulation tissue, and the formation of fibrous tissue at various time points after surgery. By comparing these factors, we aim to determine whether chlorhexidine-coated silk sutures offer any clear advantages or disadvantages in terms of tissue compatibility and overall healing.

## Materials and Methods

**Figure-1:**
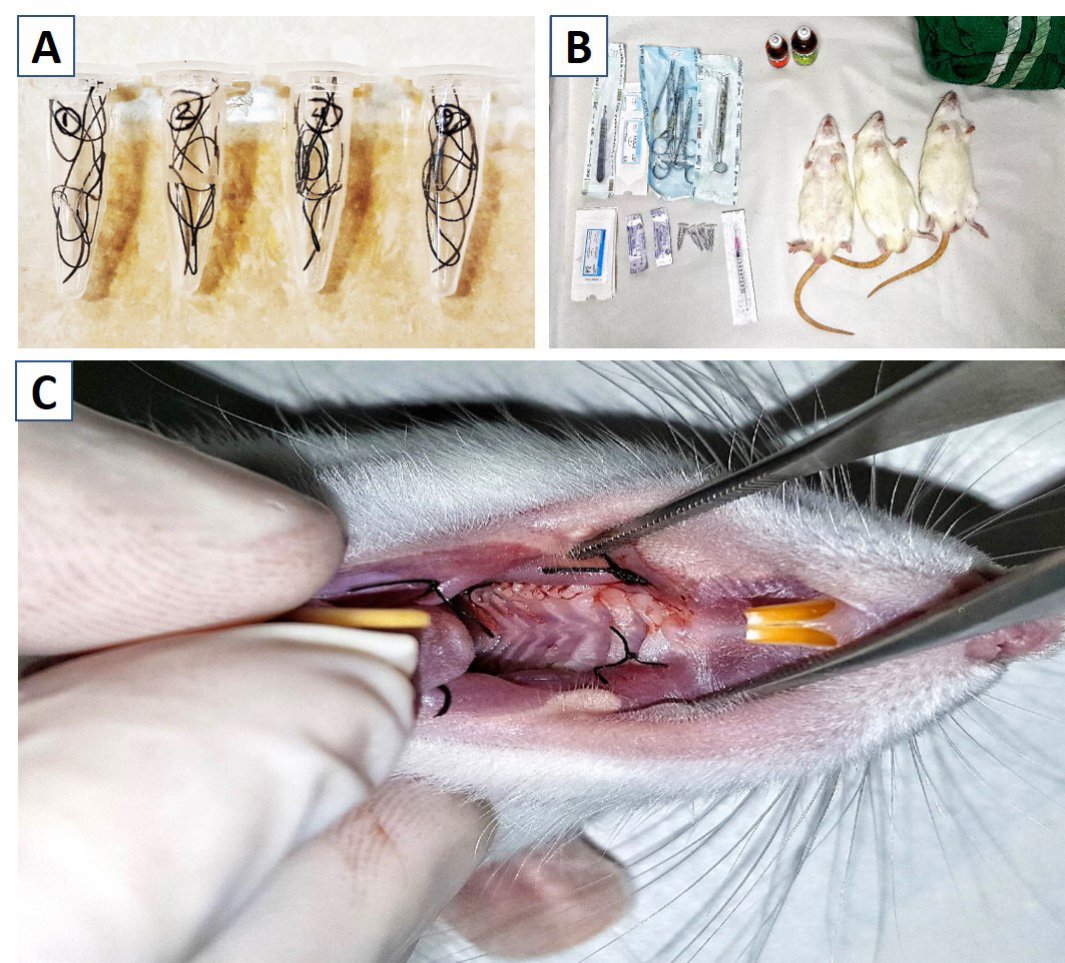
Figure 1. Preparation and Suturing Procedure. (A) Silk sutures immersed in the coating solution. (B) Anesthesia and preparation of animals alongside surgical tools. (C) Suturing performed between the upper incisors and molars.

### Suture preparation

The chlorhexidine-coated sutures were prepared at the Nano Laboratory, School of Pharmacy, Shiraz University of Medical Sciences. For this process, 4-0 silk sutures (Supasil, Supa Medical Devices, Tehran, Iran) were selected. To create the coating solution, a blend of Eudragit RL, triethyl citrate (a plasticizer), glycerol monostearate, and polyethylene glycol 4000 was mixed with isopropanol and acetone in a 3:5 ratio. Gradually, 3 cc of 3% chlorhexidine was added to 97 cc of this mixture using a shaker set at 800-900 RPM. Eighteen 25 cm lengths of 4-0 silk sutures were then cut and soaked in an ethanolic KOH solution. After thorough immersion, the sutures were removed, dried, and weighed. They were then submerged in the prepared antimicrobial coating for 10 minutes, with the container covered to prevent evaporation (Figure-1A and B). Once removed and dried, the sutures were reweighed. This dipping process was repeated 6 to 10 times, until the sutures reached a stable weight, indicating complete coating. Finally, the sutures were sterilized under UV light for 1 hour to ensure they were free from contaminants [15]. The concentration of chlorhexidine is 0.09%, which is equivalent to 900 µg per ml. In our experiment, 18 sutures, each 25 cm long (totaling 450 cm), were soaked in this solution. Therefore, the dosage applied to the sutures is 900 µg distributed across 450 cm, resulting in a concentration of 2 µg/cm. Based on this explanation, we used 2 µg/cm of chlorhexidine, which is below the cytotoxicity threshold according to ISO 10993-5 [16].

### Animals and grouping

Eighteen male Sprague-Dawley rats (10 weeks old, 200±20 g) were purchased and housed in type III polypropylene cages. The rats were kept in a room with a 12-hour light/dark cycle at a standard temperature of 23 ± 1ºC. Water was provided ad libitum. After the suturing procedure, the rats were randomly divided into three groups of six. The first group was euthanized on the third day post-surgery. The second group followed the same procedure on the fifth day, and the third group on the seventh day. The rats were euthanized using CO2 asphyxiation, a method involving the gradual introduction of carbon dioxide into a chamber to minimize distress and ensure a humane death [17].

### Incision and suturing

All 18 rats underwent mouth surgery and one side of the mouth received a coated silk suture, while the opposite side was sutured with an uncoated silk suture. To ensure consistency and minimize external variables, all sutures were purchased from the same commercial brand and applied using identical needles. Anesthesia was administered through an intramuscular injection of 10% ketamine hydrochloride (100 mg/kg), combined with 2% xylazine (10 mg/kg). Once the rats were fully anesthetized, their mouths were gently held open with gauze. A 5 mm incision was made in the keratinized gingiva between the upper incisors and molars using a No. 15 surgical blade. The incision on the left side of the upper jaw was closed with 4-0 silk sutures coated with chlorhexidine, while the right side was sutured with uncoated 4-0 silk sutures. Standard interrupted simple knots were tied with care, avoiding any tension on the tissue (Figure-1C). To maintain the integrity of the study, all sutures were placed using a 3/8 circle reverse cutting stainless steel needle.

### Sampling and Histopathological evaluation

Tissue samples were taken from the keratinized maxillary gingiva. A No.15 surgical blade was used for the excision. These samples were then immersed in a 10% formalin solution, using a volume three times that of the samples themselves, and left for 3 days. After fixation, they were sent to the pathology department at the School of Dentistry, Shiraz University of Medical Sciences for further analysis. Tissue processing and staining were done using conventional methods. Briefly, tissue samples were fixed in 10% formalin for 24 hours, then dehydrated through a graded series of ethanol (70%, 80%, 95%, 100%), cleared in xylene, and embedded in paraffin wax. Sections of 10 µm were cut, mounted on glass slides. For H&E staining, sections were stained with hematoxylin for 5-10 minutes, rinsed, differentiated, then stained with eosin for 1-2 minutes, rinsed, and dehydrated. Finally, coverslips were applied with mounting medium and allowed to dry. Slides were assessed using a grading system described in the study by Paknejad et al. [18]. The grading criteria are as follows:

Score 0: No inflammation; no histopathological evidence of inflammation.

Score 1: Mild inflammation; minimal infiltration of leukocytes into the connective tissue, with cells primarily lymphocytes and macrophages.

Score 2: Moderate inflammation; increased leukocyte infiltration, along with a rise in the number and diameter of blood vessels in the connective tissue. Cells include predominantly lymphocytes, macrophages, plasma cells, and a few neutrophils.

Score 3: Severe inflammation; marked leukocyte infiltration and a significant increase in the number and diameter of blood vessels. Cells are primarily lymphocytes, macrophages, plasma cells, and neutrophils.

Additionally, the presence or absence of granulation tissue and fibrosis was recorded, with 0 indicating absence and 1 indicating presence. Granulation tissue formation is considered an early inflammatory response, while fibrosis is a response observed in the later stages of inflammation.

### Ethical consideration

This investigation was performed in accordance with relevant guidelines and regulations of animal studies of the Ethical Committee of Shiraz University of Medical Science (ID: IR.SUMS.DENTAL.REC.1398.44).

### Statistical analysis

The Kruskal-Wallis test was used to compare inflammation scores, while the chi-square test assessed granulation and fibrous tissue formation. Graphs were created with GraphPad Prism version 8 (GraphPad Software, La Jolla, USA). A significance level of P<0.05 was set to determine statistical significance.

### Funding

The research presented in this article was supported by funding from the Shiraz University of medical science (Grant numbers: IR.SUMS.DENTAL.REC.1398.44). The funders had no role in study design, data collection and analysis, decision to publish, or preparation of the manuscript.

## Results

**Figure-2:**
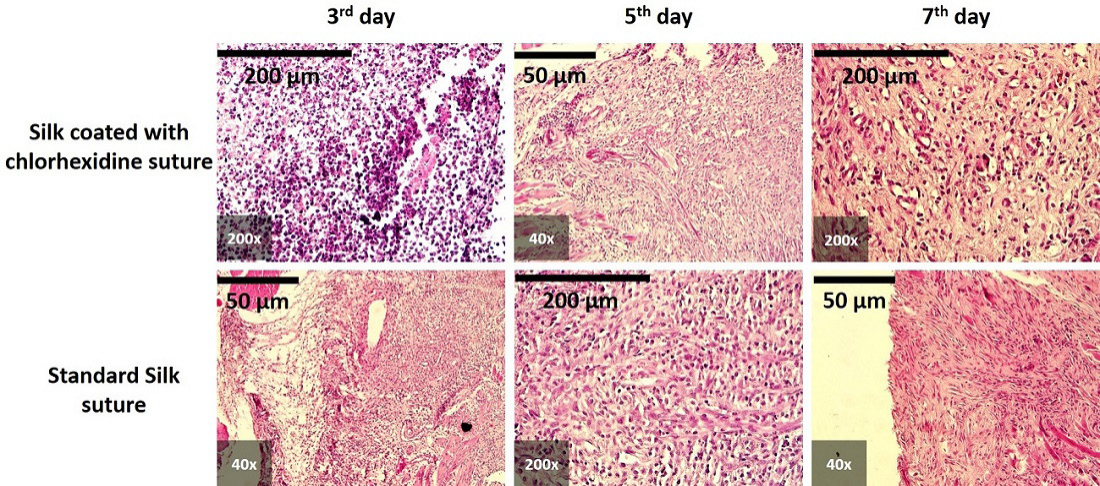
Figure 2. Histological report showing the comparison of postoperative oral incisions sutured with chlorhexidine-coated silk (top row) and standard silk sutures at 3, 5, and 7 days. In all samples, inflammatory features were evident, as shown in micrographs at different magnifications (200x and 40x). Scale bars: 200 μm and 50 μm

**Figure-3:**
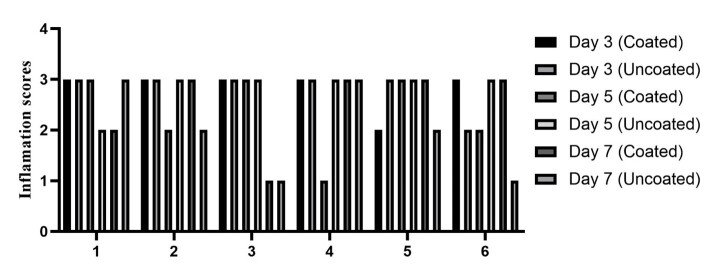
Figure 3. nflamation score in three different time point (3rd, 5th, and 7th). x-axis represent the rats: There is no significant difference between groups.

### Tissue Inflammation

All histopathological images are presented in Figure-2. On the third day, tissue response primarily indicated severe inflammation (score 3) in both the chlorhexidine-coated and non-coated groups. By the fifth day, inflammation in the coated suture group varied from mild (score 1) to severe (score 3), while the non-coated group predominantly exhibited severe inflammation (score 3). By the seventh day, both groups showed a range of inflammatory responses, from mild (score 1) to severe (score 3).

Comparing the mean and standard deviation of the tissue inflammation index, along with the results from the Kruskal-Wallis test, no significant difference was found in tissue inflammation between the regular silk suture and the chlorhexidine-coated silk suture on days three, five, or seven, with significance set at P<0.05 (Figure-3).

### Granulation and fibrous tissue formation

**Figure-4:**
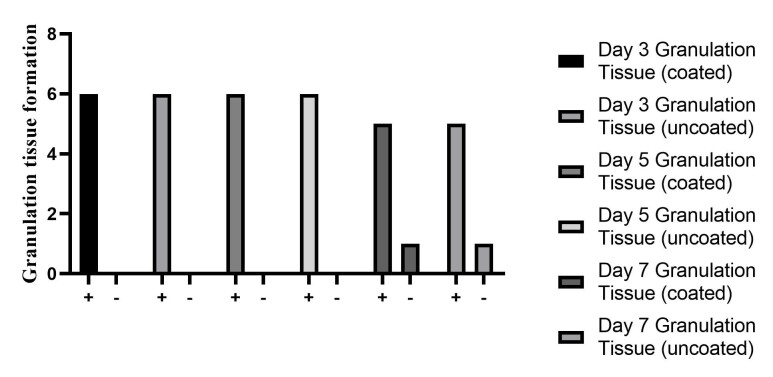
Figure 4. Granulation tissue formation analysis using chi-square: There is no significant difference between groups.

On the third and fifth days, all samples exhibited granulation tissue, with no signs of fibrous tissue formation. By the seventh day, fibrous tissue was observed in only one sample from each group, both coated and non-coated. Based on data comparison and Chi-Square test results, no significant difference was found in the formation of granulation and fibrous tissue between the regular silk suture and the chlorhexidine-coated silk suture on the third, fifth, or seventh days (Figure-4and5).

## Discussion

**Figure-5:**
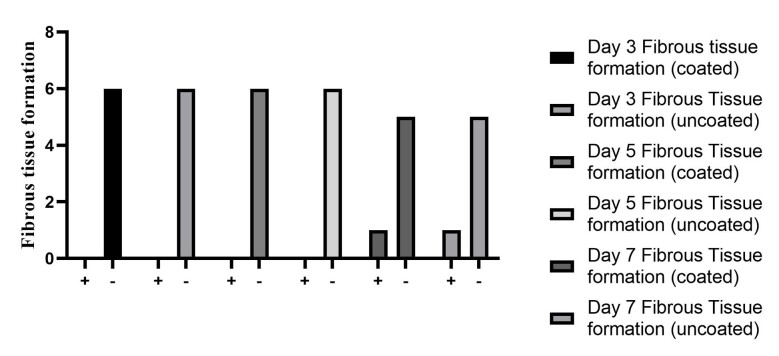
Figure 5. Fibrous tissue formation: No significant difference was observed between groups or across different time points.

Surgical site infections represent a commonly recognized complication ensuing from surgical interventions on various parts of the body. Certainly, they not only endanger the patient’s health and impede healing at the surgical site but also contribute to rising treatment costs. Most often, surgical site infections start at the incision site [19].

A significant risk factor for surgical site infections relates to the presence of foreign bodies at the surgical site, such as sutures [19]. Multifilament sutures, in particular, are more likely to predispose a patient to a surgical site infection due to their fluid-absorbing characteristics [20]. To limit surgical site infections, suture thread coatings containing antimicrobial agents, like chlorhexidine, have been used and shown good efficacy, particularly in the mouth [21]. Medical silk is known for promoting an exaggerated inflammatory response to pathogens, due to its molecular characteristics [5]. This could be especially exaggerated in the high bacterial load of the oral cavity. Microscopic analysis of silk sutures in the oral cavity identified a high volume of aerobic bacteria including Streptococcus viridans, staphylococci, and Corynebacterium [5]. Altogether, these factors increase the likelihood of surgical inflammatory complications in the oral cavity. Despite this, silk sutures are still widely used due to their favorable handling characteristics [4].

In this study, we use silk sutures coated with chlorhexidine to investigate their inflammatory tissue response in incisions made during oral surgery in male rats.

In our investigation, we did not observe any significant difference in regards to inflammatory cells between sutured with standard silk sutures versus incisions sutured with coated silk sutures at different time points, demonstrating that chlorhexidine coating does not cause tissue inflammation. In one study conducted by Xavier et al (2022), compared chlorhexidine-coated polyglycolic acid sutures to silk sutures and evaluated effects during third molar surgery. The results showed that while both groups experienced similar levels of postoperative pain, the group using chlorhexidine-coated sutures had no signs of inflammation, whereas the silk suture group had two cases of surgical site inflammation and three cases of dry sockets [21]. This result does not align with our study, where no significant difference was observed in inflammatory cells between standard and coated sutures and both show high inflammation. Chlorhexidine exhibits dose-dependent antibacterial activity. Some studies suggest that using a 4% v/v chlorhexidine coating on sutures can effectively combat Staphylococcus aureus, Staphylococcus epidermidis, and Escherichia coli [22]. However, higher concentrations may induce cytotoxicity [23]. To mitigate this risk, we opted for a 0.09% chlorhexidine coating solution, which is lower than the concentration typically effective against bacteria. The Xavier’s study lacks information on the dose of chlorhexidine used in coating the silk sutures, which contributes to the discrepancy in results. Additionally, differences in methods for evaluating inflammation between our study and Xavier’s could also account for the variation in findings.

Fibrous and granulation tissue formation in our study showed no significant difference, implying that using this concentration of chlorhexidine-coated silk sutures does not trigger any adverse tissue reactions. The way chlorhexidine is released into the physiological environment is also crucial. One study investigated the effects of chlorhexidine-containing substances as intracanal medicaments on subcutaneous connective tissue in mice. Their histopathological investigation revealed that the tissue response varied with the concentration of chlorhexidine used. Specifically, Calen paste with 0.5% chlorhexidine led to a reparative tissue response, while Calen paste with 2% chlorhexidine and a 2% chlorhexidine gel induced a persistent inflammatory response. Notably, among the higher concentrations, the 2% chlorhexidine gel caused a more intense inflammatory reaction compared to the 2% chlorhexidine in Calen paste. This underscores the importance of slow-release properties in reducing the risk of adverse tissue reactions [24]. In this regard, many studies employ different techniques to enhance their materials by using carboxymethyl cellulose gel or fatty acid carriers [16][25]. In this study, we used the lowest concentration of chlorhexidine solution with multiple dippings of the thread in a solution containing Eudragit RL to enhance its slow-release properties.

In terms of wound healing, no differences were observed in granulation and fibrous tissue formation between the coated and uncoated groups. While in vitro studies indicate that chlorhexidine exhibits high cytotoxicity against fibroblasts and keratinocytes [13][23], it appears to be safe for use in surgical fields. However, caution should be exercised in selecting the concentration. Other studies also show similar results, suggesting that coating surgical sutures with antibacterial materials either has no effect or only a slight improvement on wound healing [26][27].

Additionally, the lack of difference observed between standard silk sutures and those coated with chlorhexidine in terms of granulation and fibrous tissue formation indicates that the concentration of chlorhexidine used does not have a cytotoxic effect and does not alter the healing process. However, its antibacterial efficacy and release behavior should be further evaluated.

## Conclusion

In this study, we concentrated on the impacts of chlorhexidine coated silk sutures with respect to tissue inflammation, granulation, and fibrous tissue formation. Inflammation and healing were not significantly different between the two suture types, suggesting that this concentration does not cause any adverse tissue reactions.

### Declaration of Generative AI

During the preparation of this work, the authors used ChatGPT 3.5 to correct the grammar, punctuation, and syntax, as well as to improve the clarity of the texts. The authors reviewed and edited the content as needed and took full responsibility for the content of the publication.

## Conflict of Interest

The authors declared no potential conflicts of interest with respect to the research, authorship, and/or publication of this article.

## References

[R1] Lekic N, Dodds SD (2022). Suture Materials, Needles, and Methods of Skin Closure: What
Every Hand Surgeon Should Know. The Journal of Hand Surgery.

[R2] Javed F, Al-Askar M, Almas K, Romanos GE, Al-Hezaimi K (2012). Tissue reactions to various suture materials used in oral
surgical interventions. ISRN Dent.

[R3] Suthar P, Shah S, Waknis P, Limaye G, Saha A, Sathe P (2020). Comparing intra-oral wound healing after alveoloplasty using silk
sutures and n-butyl-2-cyanoacrylate. Journal of the Korean Association of Oral and Maxillofacial Surgeons.

[R4] Faris A, Khalid L, Hashim M, Yaghi S, Magde T, Bouresly W, et al (2022). Characteristics of Suture Materials Used in Oral Surgery:
Systematic Review. International Dental Journal.

[R5] Sortino F, Lombardo C, Sciacca A (2008). Silk and polyglycolic acid in oral surgery: a comparative study. Oral Surgery, Oral Medicine, Oral Pathology, Oral Radiology, and
Endodontology.

[R6] Rakhmatullayeva D, Ospanova A, Bekissanova Z, Jumagaziyeva A, Savdenbekova B, Seidulayeva A, et al (2023). Development and characterization of antibacterial coatings on
surgical sutures based on sodium carboxymethyl
cellulose/chitosan/chlorhexidine. Int J Biol Macromol.

[R7] Collins JR, Veras K, Hernández M, Hou W, Hong H, Romanos GE (2021). Anti-inflammatory effect of salt water and chlorhexidine 0.12%
mouthrinse after periodontal surgery: a randomized prospective clinical
study. Clin Oral Investig.

[R8] Brookes ZLS, Bescos R, Belfield LA, Ali K, Roberts A (2020). Current uses of chlorhexidine for management of oral disease: a
narrative review. Journal of Dentistry.

[R9] Krishnan S, Periasamy S, Murugaiyan A (2020). Comparing the efficacy of triclosan coated sutures versus
chlorhexidine coated sutures in preventing surgical site infection after
removal of impacted mandibular third molar. Journal of Pharmaceutical Research International.

[R10] Sethi KS, Karde PA, Joshi CP (2016). Comparative evaluation of sutures coated with triclosan and
chlorhexidine for oral biofilm inhibition potential and antimicrobial
activity against periodontal pathogens: An: in vitro: study. Indian Journal of Dental Research.

[R11] Chaganti S, Kunthsam V, Velangini SY, Alzahrani KJ, Alzahrani FM, Halawani IF, et al (2023). Comparison of bacterial colonization on absorbable non-coated
suture with Triclosan- or Chlorhexidine-coated sutures: a randomized
controlled study. Eur Rev Med Pharmacol Sci.

[R12] Main RC (2008). Should chlorhexidine gluconate be used in wound cleansing. J Wound Care.

[R13] Tatnall FM, Leigh IM, Gibson JR (1990). Comparative study of antiseptic toxicity on basal keratinocytes,
transformed human keratinocytes and fibroblasts. Skin Pharmacol.

[R14] Dennis C, Sethu S, Nayak S, Mohan L, Morsi YY, Manivasagam G (2016). Suture materials - Current and emerging trends. J Biomed Mater Res A.

[R15] Ammar HO, Ghorab MM, Felton LA, Gad S, Fouly AA (2016). Effect of Antiadherents on the Physical and Drug Release
Properties of Acrylic Polymeric Films. AAPS PharmSciTech.

[R16] Obermeier A, Schneider J, Wehner S, Matl FD, Schieker M, von Eisenhart-Rothe, et al (2014). Novel high efficient coatings for anti-microbial surgical sutures
using chlorhexidine in fatty acid slow-release carrier systems. PLoS One.

[R17] Underwood W, Anthony R (2020). AVMA guidelines for the euthanasia of animals: 2020 edition. Retrieved on March.

[R18] Paknejad M, Bayani M, Yaghobee S, Kharazifard MJ, Jahedmanesh N (2015). Histopathological evaluation of gingival tissue overlying
two-stage implants after placement of cover screws. Biotechnology & Biotechnological Equipment.

[R19] Evrosimovska B (2021). Types of suturing materials in oral surgery. International Journal of Business and Technology.

[R20] Chua RA, Lim SK, Chee CF, Chin SP, Kiew LV, Sim KS, Tay ST (2022 Feb 1). Surgical site infection and development of antimicrobial sutures: a review. European Review for Medical & Pharmacological Sciences.

[R21] Xavier SA, Wahab A (2022). Comparison of chlorhexidine coated polyglycolic acid sutures with
silk sutures during third molar surgery: A prospective, randomized,
double-blind clinical study. International journal of health sciences.

[R22] Walker G, Rude M, Cirillo SLG, Cirillo JD (2009). Efficacy of using sutures treated with povidone-iodine or
chlorhexidine for preventing growth of Staphylococcus and Escherichia coli. Plast Reconstr Surg.

[R23] Dinu S, Matichescu A, Buzatu R, Marcovici I, Geamantan-Sirbu A, Semenescu AD, et al (2024). Insights into the Cytotoxicity and Irritant Potential of
Chlorhexidine Digluconate: An In Vitro and In Ovo Safety Screening. Dentistry Journal.

[R24] Pereira MS, Faria G, Bezerra da, Tanomaru-Filho M, Kuga MC, Rossi MA (2012). Response of mice connective tissue to intracanal dressings
containing chlorhexidine. Microsc Res Tech.

[R25] Pérez-Köhler B, Benito-Martínez S, Rodríguez M, García-Moreno F, Pascual G, Bellón JM (2019). Experimental study on the use of a chlorhexidine-loaded
carboxymethylcellulose gel as antibacterial coating for hernia repair meshes. Hernia.

[R26] Karde PA, Sethi KS, Mahale SA, Mamajiwala AS, Kale AM, Joshi CP (2019). Comparative evaluation of two antibacterial-coated resorbable
sutures versus noncoated resorbable sutures in periodontal flap surgery: A
clinico-microbiological study. Journal of Indian Society of Periodontology.

[R27] Sharma C, Rajiv N, Galgali SR (2017). Microbial adherence on 2 different suture materials in patients
undergoing periodontal flap surgery–A pilot study. J Med Sci Clin Res.

